# Randomized comparison of fibrinogen concentrate versus cryoprecipitate for bleeding control in pediatric cardiac surgery (FICCS study)

**DOI:** 10.1186/cc11045

**Published:** 2012-03-20

**Authors:** F Galas, L Hajjar, B Sorensen, J Almeida, M Sundin, V Guimaraes, S Zefferino, L Camara, F Maua, M Moreira, C Puttini, M Carmona, J Auler, R Nakamura

**Affiliations:** 1 Heart Institute, São Paulo, Brazil; 2 Guy's and St Thomas' NHS Foundation Trust & King's College London School of Medicine, London, UK

## Introduction

We compared hemostatic outcomes after treatment with fibrinogen concentrate or cryoprecipitate in pediatric cardiac surgery patients with intraoperative bleeding.

## Methods

This was a single-center, randomized, open-label study. Key inclusion criteria were age <15 years, cardiac surgery involving cardiopulmonary bypass, intraoperative bleeding after neutralization of heparin, and hypofibrinogenemia. Patients received fibrinogen concentrate (60 mg/kg body weight; Haemocomplettan^® ^P) or cryoprecipitate (10 ml/kg body weight). After study medication, allogeneic blood products were administered as required. Blood samples taken immediately before randomization and 1, 24 and 48 hours after study medication were subjected to laboratory and thromboelastometry (ROTEM) coagulation tests.

## Results

Sixty-three patients (fibrinogen concentrate: 30; cryoprecipitate: 33) completed the study. The median age was 3 years 5 months and the median weight was 6.7 kg. Median fibrinogen doses were 504 mg (fibrinogen concentrate) and 402 mg (cryoprecipitate). Plasma fibrinogen concentrations increased after study medication and were similar in the two groups. No significant between-group differences were observed in PT, aPTT or platelet count. In both groups, all ROTEM parameters showed significant improvement after study medication, with no clinically relevant between-group differences in any of the EXTEM, INTEM or FIBTEM clotting parameters. Total avoidance of allogeneic blood product transfusion was achieved in 70% of patients in the fibrinogen concentrate group versus 18.2% in the cryoprecipitate group (*P *< 0.001). The mean bleeding mass was significantly lower in the fibrinogen concentrate group than in the cryoprecipitate group after 30 minutes. The thorax was opened after study medication in zero patients (0%) in the fibrinogen concentrate group and in six patients (18.2%) in the cryoprecipitate group (*P *= 0.025).

## Conclusion

Fibrinogen concentrate raised fibrinogen levels and improved coagulation measures to a similar degree as cryoprecipitate. Bleeding and transfusion of allogeneic blood products were lower in the fibrinogen concentrate group. Fibrinogen concentrate may be a valuable option for controlling bleeding and avoiding transfusion in cardiac surgery.

